# A computational model of language functions in flexible goal-directed behaviour

**DOI:** 10.1038/s41598-020-78252-y

**Published:** 2020-12-10

**Authors:** Giovanni Granato, Anna M. Borghi, Gianluca Baldassarre

**Affiliations:** 1 Laboratory of Computational Embodied Neuroscience, Institute of Cognitive Sciences and Technologies, National Research Council of Italy, Rome, Italy; 2 Department of Dynamic and Clinical Psychology, Sapienza University of Rome, Institute of Cognitive Sciences and Technologies, National Research Council of Italy, Rome, Italy

**Keywords:** Psychology, Cognitive neuroscience, Computational neuroscience

## Abstract

The function of language in high-order goal-directed human cognition is an important topic at the centre of current debates. Experimental evidence shows that inner speech, representing a self-directed form of language, empowers cognitive processes such as working memory, perception, categorization, and executive functions. Here we study the relations between inner speech and processes like feedback processing and cognitive flexibility. To this aim we propose a computational model that controls an artificial agent who uses inner speech to internally manipulate its representations. The agent is able to reproduce human behavioural data collected during the solution of the Wisconsin Card Sorting test, a neuropsychological test measuring cognitive flexibility, both in the basic condition and when a verbal shadowing protocol is used. The components of the model were systematically lesioned to clarify the specific impact of inner speech on the agent’s behaviour. The results indicate that inner speech improves the efficiency of internal representation manipulation. Specifically, it makes the representations linked to specific visual features more disentangled, thus improving the agent’s capacity to engage/disengage attention on stimulus features after positive/negative action outcomes. Overall, the model shows how inner speech could improve goal-directed internal manipulation of representations and enhance behavioural flexibility.

## Introduction

The relationship between language and cognition is a long-standing issue at the centre of theoretical debate and scientific research. Recent years have seen the proposal of embodied and grounded views of cognition, according to which language is grounded in perception, action, and emotional systems (e.g.^1–5^). A wealth of evidence shows that processing manipulable nouns and action verbs activates sensorimotor representations. Compelling results demonstrate that concepts and words are grounded in sensorimotor and affective systems. However, sometimes the focus on embodiment has led to neglect important effects of language on cognition. Within embodied cognition approaches, words have indeed been mainly intended as pointers to their referents—objects and entities in the world (e.g., see^6^ and^7^ for a computational model, and^8^ for critiques). Although this is undoubtedly an important role of words, language also plays other relevant functions for high-order cognition.

In recent years, new trends of research are ascribing to language a richer set of functionalities. First, embodied perspectives have been recently flanked by multiple representation views^9,10^, that recognise the importance not only of sensorimotor processes but also of emotional/affective and linguistic experiences in grounding cognition. The embodied/grounded approach, that ascribes a major role to the sensorimotor grounding of concepts and words, was traditionally in contrast with statistical/distributional views according to which meaning can be captured through linguistic associations. In recent years, hybrid models are emerging that ascribe importance to both sensorimotor and linguistic experience, combining the embodied/grounded perspective with the insights deriving from statistical distributional approaches^11,12^. For example, the consensus is growing on the idea that abstract concepts such as “freedom” and “truth” are grounded not only in sensorimotor and emotional experience but also in social and linguistic elements^13,14^.

Second, an increasing number of studies are showing that language impacts cognition in multiple ways. These studies highlight how language influences not only high-level cognition (e.g., reasoning and problem solving) but also perception, for example helping humans to better recognise and categorise objects and entities^15,16^.

Third, neo-whorfian approaches have reopened the debate about the idea that not only language in general but also the specific language we speak shapes the way we think^17,18^. Thus a growing amount of evidence is showing that different spoken languages influence the way we perceive time (e.g.^19,20^), colour (e.g.^21,22^, odour (e.g.^23^), and pitch (e.g.^24^).

Language has also been proposed to be conceived as a sort of *tool*. First, in social interactions words can be used similarly to physical tools to extend the bodily space in relation to object reaching^25^. Second, words can also work as cognitive tools to affect our internal representations of the physical and social environment. The notion of language as a tool is not new, and it was introduced by distinguished authors as Wittgenstein^26^ and Vygotsky^27^. More recently, different authors have expanded this idea in different but similar directions (e.g.^28–31^). In particular, it has been highlighted that language can act as a *cognitive tool*. Language, both in its overt form of spoken utterances and in its covert form as *inner speech*, can ameliorate our thought processes, extend our memory, and support our prediction capabilities^9,32,33^.

The notion of inner speech has a long history and is hotly debated in the recent literature (for reviews, see^34,35^). Inner speech was first introduced by Vygotsky who proposed that it results from a developmental process leading to the progressive internalization of overt speech. Importantly for this work, the notion of inner speech has been later used in the context of working memory, in particular by stressing its role as a rehearsal mechanism that actively maintains information to support planning processes^36^. Current research focuses on different functions that inner speech might have, in particular in relation to cognitive control^34^ representing a relevant issue also investigated here. Moreover, recent literature has attested the existence of different kinds of inner speech, such as wilful/deliberative^37^ vs. spontaneous inner speech, condensed vs. expanded inner speech, monologic vs. dialogic inner speech, and evaluative/motivational inner speech^35^. Importantly for this work, some authors have highlighted the relationship of inner speech with second order cognition and metacognition^38^ and recent studies have investigated the relation between metacognition and the strategy changes adopted following error detection^39^. Despite these recent advancements on the impact of overt and covert language on cognition, the internal mechanisms supporting these processes are not fully clear and computational models as the one proposed here could support their identification.

In this work we focus in particular on the interaction between language and cognition within the framework of *embodied goal-directed behaviour*. This refers to a set of behaviours and underlying cognitive processes where action is guided by a goal to accomplish rather than by stimuli directly triggering thoughts and behaviours^40,41^. To this purpose, we present here a neuro-inspired computational model able to accomplish a desired overall goal by scheduling sub-goals and actions. Importantly, the model is able to pursue them on the basis of the internal manipulation of representations supported by language (“Methods” section). The model extends a previous model^42^ formed by multiple neuro-inspired components with the addition of a critical inner-speech component, allowing the model to account for the results of experiments where humans might use inner speech to manipulate internal representations. The kind of inner speech we model is monologic (as the model does not interact with other agents) and deliberative/wilful (as supporting decision making). The model inner-speech component can be considered a form of metacognition—the system represents the chosen decision and evaluates it either positively or negatively, and this influences the strategy it will follow. In particular, the component strengthens working memory contents, as the phono-articulatory loop in Baddeley’s theory^43^, and plays a motivational role, as proposed by different authors who stress the motivational and self-reinforcing role of inner speech^44,45^. In our study we compared the model behaviour with the performance of human participants solving the Wisconsin Cards Sorting test (WCST), an important neuropsychological test directed to measure cognitive flexibility^46^ (“Methods” section). In particular, we used here a version of the WCST involving *verbal shadowing*, an experimental protocol causing the interference with inner speech; to this purpose we considered the dataset previously published in^47^. Moreover, we studied the internal functioning of the model and the effects of focused lesions of some of its cognitive and linguistic components. The results show the specific effects that inner speech might have on the internal manipulation of representations (section "Results"). As discussed in sections "Discussion" and "Conclusions", the model thus represents an operational hypothesis on how language might enhance flexible goal-directed behaviour.

## Methods

This section first illustrates the neuropsychological task used to test the model. This task is based on an experimental protocol integrating the WCST and motor and verbal shadowing protocols^47^. Then the section illustrates the functional and computational details of the model.

### Task and experimental conditions

**Figure 1:**
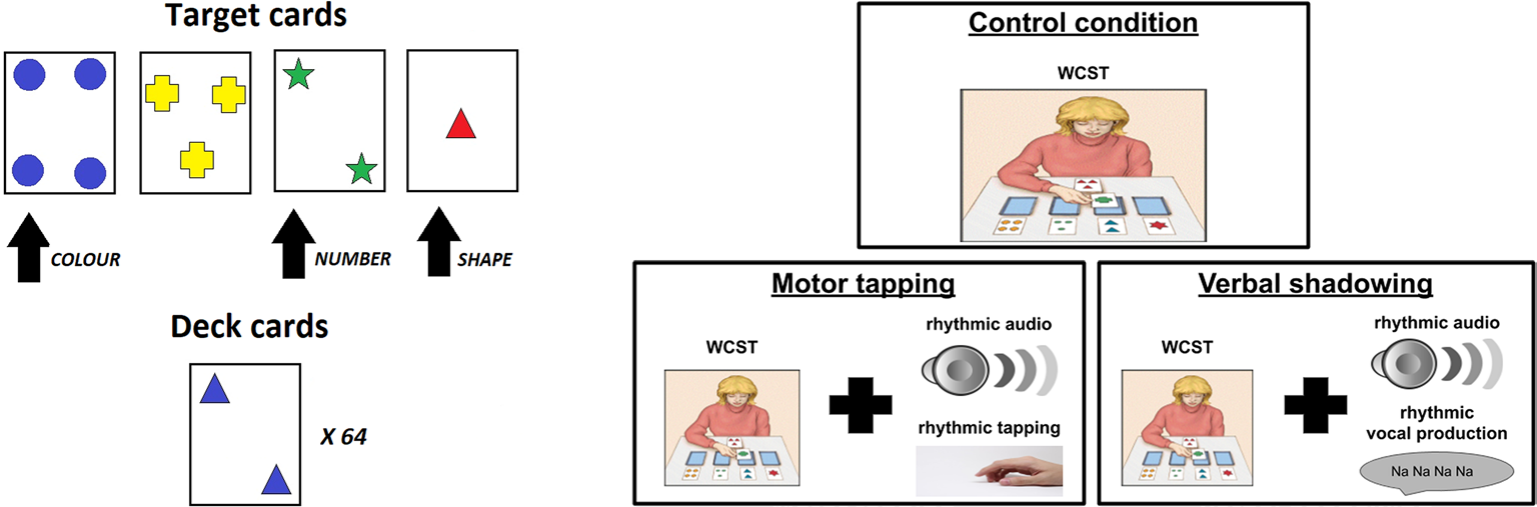
Left: WCST setting; the black arrows indicate the target cards that correspond to a certain category that matches the category of the shown deck card. Right: Experimental protocols used to test the model, involving the basic WCST (control), and a WCST where the participant has to perform a rhythmic tapping following a rhythmic audio, and a critical analogous verbal-shadowing condition affecting inner speech.

The Wisconsin Cards Sorting Test (WCST)^46^ is a neuropsychological test allowing the measure of executive functions, in particular of cognitive flexibility, that is, the capacity of rapidly changing behaviour on the basis of an external feedback to achieve a target goal^48^. The WCST is very sensitive to frontal lesions that cause perseverative behaviours^46,49^. The task (Fig. 1, top) involves a deck of 64 cards and four ‘target cards’ set on a table. Each deck card contains a specific combination of geometrical shapes varying with respect to three categories (colour, shape, number), each characterised by four attributes (respectively: red, green, blue, yellow; stars, triangles, circles, crosses; one, two, three, four). Each target card presents a unique combination of attributes not shared with the other three target cards. Participants draw the deck cards one after the other, and are required to sort each of them choosing one of the three categories. In order to choose a category participants have to put the drawn deck card under one target-card matching it for the attribute of the chosen category (e.g., if the chosen category is ‘colour’, a card with a yellow item has to be put under the target card with a yellow item). The correct category (sorting rule) is unknown by the participant. After each sorting attempt the experimenter provides a feedback (‘correct’ or ‘not correct’) depending on the current sorting rule and the participant has to infer this rule on the basis of the feedback. After a fixed number of succeeding correct matches the experimenter changes the correct sorting rule without telling the participant who has thus to adapt to the new rule by inferring it on the basis of the feedback.

The model performance was compared with human performance during the solution of WCST coupled to the shadowing protocols, in particular the model was compared with the dataset previously published in^47^. Human participants were psychology college students (average age: 20 years). The protocol used included three experimental conditions (Fig. 1, bottom): control condition—participants solved the basic WCST; motor tapping—participants solved the WCST while executing a finger tapping task following a rhythmic sound; verbal shadowing—participants solved the WCST while vocally repeating the sound ‘Na, Na, ...’ following a rhythmic sound. The authors report that the participants who solved the WCST during the verbal shadowing protocol exhibit a behavioural impairment (i.e., an increase of behavioural errors, see below) compared with those that solved the WCST with no interfering protocol (control condition). The authors also reported an impairment in the participants that solved the WCST during the motor-tapping protocol, but this was lighter than in the verbal shadowing condition. The authors interpreted this outcome by suggesting that inner-speech, together with other processes such as attention or working memory, is a cognitive support for problem solving processes. Furthermore, also based on cross-cultural data, they hypothesised that there are individual differences with respect to inner-speech use during problem solving processes.

The model scoring followed the official documentation^46^ and was based on five principal behavioural indices giving a full behavioural and cognitive profile of the test performance: CC—*Completed Categories*, indicating the number, out of six, of the performed non-interrupted ten-card sequences of correct sorting; TE—*Total Errors*, indicating the global performance/deficit; PE—*Perseverative Errors*, indicating a perseverative behaviour; NPE—*Non Perseverative Errors*, indicating an attentional failure or an incorrect inferential reasoning; FMS—*Failure to Maintain Set*, indicating a distracted behaviour.

### Model

**Figure 2:**
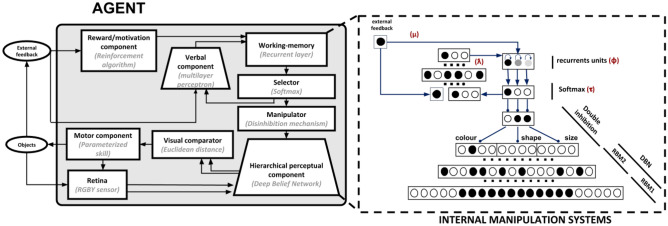
Architecture of the model. Left: components of the model. Right: zoom on the neural-network components of the model performing the internal manipulation of representations aided by the language component. The red symbols near the components identify model parameters important for specific cognitive functions (see text for details).

The model, which builds on a previous model^42^, is formed by neuro-inspired components that support a number of functions needed to support goal-directed behaviour (Fig. 2). The components are these: (a) Visual sensor: this component gathers visual information from deck cards and target cards, analogously to the eye retina; (b) Hierarchical perceptual component: this component extracts input visual features at increasing levels of abstraction, analogously to the visual brain system^50^; if activated by top-down mechanisms, this component can also re-generate relevant aspects of percepts, for example based on imagination mechanisms^51^; (c) Abstract working memory: this component stores the task sub-goals (sorting rules) chosen by the model, a function that in the brain is mostly supported by frontal cortices^48,52^; (d) Motivational system: this component uses the external feedback to update the information in working memory, a function that in the brain is mostly supported by ventral basal ganglia^53,54^; (e) Selector: this component chooses a sorting rule and biases the perceptual system (manipulation of internal representations), a function analogous to the top-down control that the fronto-parietal cortex, aided by basal ganglia, exerts on lower-level internal perceptual representations^55,56^; (f) Comparator: this component executes visual matching of the deck and target cards, based on the comparison of low-level perceptual representations of the cards by simulating the attentional/imagination processes of brain^42^; in the brain these processes might rely on a distributed network involving the frontal and temporal-occipital cortices^51,57^; (g) Motor system: this component controls saccades and actions displacing the deck cards close to the chosen target card.

A key further part of the model is the *inner-speech component* inspired by the brain networks that integrate linguistic and emotional information^58,59^. First, the component transmits information on the relevance of rules to the working memory, in particular information on the sub-goals (identity of the rule) whose priority should be changed, and the positive/negative valence, and intensity, of such change. Moreover, the component implements a phonological-loop storing the current rule independently of its possible pragmatic use.

### Computational details of the model

The key components of the model are implemented with neural networks whereas the ancillary components (e.g., the motor components) are implemented by reproducing their functions in an abstract fashion (Fig. 2). This section explains the functioning of the model to a level sufficient to interpret the results while the computational details of the model are reported in the Supplementary Materials. The code of the model is publicly available for online download from the GitHub  repository: https://github.com/GiovanniGranato/Flexible-goal-directed-behaviour-and-Inner-Speech.

The working memory is formed by three units with recurrent self-synapses. The self-activation decays to a baseline value (0.5) based on a decaying rate parameter $\phi$. These units, ranging in [0, 1], encode the biases for selection of the three matching rules.

The motivational component is supported by a reinforcement mechanism that increases or decreases the activation of the working-memory recurrent units. In particular, the component increases or decreases the activation of the unit that encodes the chosen sorting rule by an amount related to a parameter $\mu$.

The hierarchical perceptual component is formed by a modified version of a deep generative neural network, in particular a *Deep Belief Network* (DBN;^60^) composed by two stacked *Restricted Boltzmann Machines* (RBM;^61^). The component can encode the input (images of either the deck or target card) at different levels of abstraction. The component can also reconstruct (generate) the relevant aspects of the input, on the basis of the activation of its high-level units, through a top-down information flow supported by the DBN bidirectional connections. To this purpose, the last inner layer of the DBN encodes 12 possible attributes, divided into three groups corresponding to the three card categories (colour, shape, size).

The working memory stores information that is processed by the selector and the manipulator. In particular, the selector receives as input the information from the working memory and uses a *soft-max* function to compute the probabilities of selecting the different matching rules (a temperature parameter $\tau$ regulates how much the differences between the bias values of the working memory are enhanced/reduced); these probabilities are then used to extract a ‘winner unit’ that receives maximum activation and encodes the chosen matching rule (this process abstracts a neural competition based on lateral inhibition).

The manipulator (see Fig. 2) is formed by three units that permanently inhibit three corresponding category units. The winner unit of the selector inhibits one unit of the manipulator, which in turn temporarily removes its inhibition from the corresponding category attribute units at the highest level of the DBN. The manipulator also implements a *hard-max* function (that abstracts inhibitory lateral connections) leading to strongly activate only one attribute unit within the three groups of 4 units representing the categories (see Fig. 2).

The language component is supported by a neural network (multi-layer perceptron) formed by an input layer, a hidden layer, and an output layer. This component receives a composite input from the selector (three units representing the chosen rule) and from the external environment (one unit representing the external feedback on the incorrect/correct matching). The output encodes the identity and valence of the last chosen rule, projecting, with one-to-one connections, to the working memory component. For example, if the model chooses the colour rule and receives a positive feedback, the language component causes an increase of the activation of the first unit of the working memory encoding the colour rule. Conversely, if the model chooses the colour rule and receives a negative feedback, the component causes a decrease of the activation of the first unit of the working memory. Analogous processes involve the working-memory form and size units in the case the system selects respectively the form and size rules. The language component is activated two times: a first time to implement a phonological loop storing linguistic information on the chosen rule; a second time to contribute to the feedback-dependent updating of the working memory content. In the first activation, the component is activated by the one-hot code from the selector, based on the current working memory content, and by a value of 1 at the level of the feedback unit (meaning ‘maintenance of the current rule’): this activation has the function of storing information on the decision rule within the phonological loop. In the second activation, taking place after the search and comparison of the deck/target cards and the action performance, the component is activated by the selector activation and by the external feedback: this activation contributes to update the working memory content in view of the next trial. The contribution of language to the working memory is regulated by a coefficient $\lambda$ ranging in [0, 1] and representing the connection weights from the language component to the working-memory units.

The remaining components, computationally simplified, are relevant to support the interaction of the agent with the simulated card environment (see^62^ for the simple simulator used to this purpose). The visual sensor is formed by an RGBY matrix that encodes a small portion of the environment, approximately covering one card per time, and represents the input following an attention-focused saccade onto one specific card. The motor component generates these saccades, in particular couples of saccades directing to the deck card and a target card, with the saccade couples involving the different target cards in sequence. Moreover, when the model detects a matching between the deck and target card it moves the deck card under the target card. Overall, the processes and behaviour of the model are as follows: (a) the working memory-selector-manipulator exert a top-down disinhibition of the attributes of the selected category; (b) the consequent card image causes the activation of one of such attributes through the bottom-up activation of the hierarchical perceptual component; (c) such attribute causes a top-down re-generation of the image pivoting on the selected attribute (e.g., if the system sees a card with a large red circle, and the selected matching rule is ‘colour’, then the model will imagine a prototypical ‘red image’, e.g. a shapeless colour blob); (d) the visual comparator gets as input the two reconstructed (selected-attribute dependent) images corresponding to the deck card and target cards, and compares them to check if they match (a matching is detected if the Euclidean distance between the pixel vectors of the two images is below a threshold); (e) in case of matching, the model displaces the deck card under the target card, otherwise it performs another couple of saccades until it finds a matching deck and target card; (f) following the card displacement, a simulated ‘external operator’ returns to the model a positive or negative feedback.

## Results

In this section, we first present the results of the comparison of the model behaviour with the human behaviour aimed to investigate the underlying cognitive processes. We then study the model functioning when different aspects of its components are lesioned. Finally, we investigate the internal dynamics of the model with a focus on the role of the language component.

### Model configurations, humans cognitive profiles and behavioural indices

We used a statistical search method based on the minimisation of the mean square error (MSE) to find the model parameters that best fit the human data in the three conditions of the WCST, namely the control, motor tapping, and verbal shadowing conditions. The parameters can be interpreted as the relative weights of the simulated cognitive traits of the model (negative feedback sensitivity, memory forgetting, distractability, language contribution), and hence of the modelled human participants. Table 1 shows the values of the parameters found with the statistical procedure, now examined in detail.

**Table 1:** Values of the parameters of the models that produce the best fit of the data on the WCST indices, for the control and experimental groups, reported in^47^).

	Error sensitivity ($\mu$)	Forgetting speed ($\phi$)	Distractibility ($\tau$)	Language contribution ($\lambda$)
Control	0.49	0.97	0.10	0.81
Motor tap.	0.17	0.09	0.12	0.23
Verbal shad.	0.14	0.14	0.13	0.14

We first focus on the parameter $\lambda$ representing the level of involvement of language processes in the solution of the task. The table shows that the contribution of language is higher in the control condition ($\lambda = 0.81$) than in the motor tapping condition ($\lambda = 0.23$) and verbal shadowing condition ($\lambda = 0.14$). The lower value in the shadowing condition corroborates the model as it indicates that in such condition the model relies on resources other than language to solve the task. The lower value in the tapping condition was instead partially unexpected because this condition should not cause a decrease of the inner-speech contribution. However, since the motor tapping condition involves an auditory process needed to follow the external rhythmic sound, we propose that this interferes with the linguistic contribution to the memory processes as involving the same integrated phonological processes. Thus the participants rely less on language and more on visual working memory and imagery relying on the non-linguistic processes of the model. However, these alternative solutions represent sub-optimal solutions for humans, used to rely on inner speech, and thus lead to a lower performance with respect to controls. This result can be be considered as a prediction of the model, possibly testable in future empirical experiments.

The control group has a very high $\phi$ value ($\phi = 0.97$). This suggests a compensating interaction between inner speech, error sensitivity and working-memory information decay. In particular, in case of a repeated strong bias toward a specific rule caused by a high error sensitivity ($\mu$ = 0.49), a low distractibility ($\tau$ = 0.10), and a high language contribution ($\lambda$ = 0.81), a large decay of working memory contents does not prevent effective decision making.

The control and experimental groups show a higher distractibility value ($\tau$) and a lower error sensitivity ($\mu$). These results can be explained by an increased cognitive load in these conditions that can cause inefficient decision-making and error detection processes.

Finally, the experimental groups appear less different from each other compared to the control group (Fig. S1, Supplementary Materials), corroborating the idea that both experimental conditions cause a performance decrease because both interfere with inner speech.

#### Statistical analysis of the relation between parameters and behavioural indices

We now present the correlation results (Pearson’s coefficients) between the model parameters, representing the strength of its cognitive processes, and the behavioural indices scored in the WCST (Table 2).

**Table 2:** Pearson’s correlations between the model key parameters ($\mu$, $\phi$, $\tau$, $\lambda$) and WCST indices.

Indices	Parameters			
	${\mu }$	${\phi }$	${\tau }$	${\lambda }$
CC	+ 0.00	− ***0.34***	− ***0.72***	*** +0.32***
TE	− *0.05*	+ ***0.40***	+ ***0.70***	− ***0.37***
PE	− *0.08*	+ ***0.40***	+ ***0.65***	− ***0.40***
NPE	− 0.03	+ ***0.40***	+ ***0.72***	− ***0.35***
FMS	+ 0.01	+ *0.04*	***+0.86***	− *0.04*

The table highlights in **bold** the correlation indexes with an absolute value above 0.3, and in *Italics* those that are statistically significant.

The $\mu$ parameter (error sensitivity) did not show a strong correlation with anyone of behavioural indexes. However, it showed statistically significant ($p<0.05$) negative correlations with PE ($r = - 0.08$) and TE ($r = - 0.05$). These correlations confirm the role of this parameter (error sensitivity) for cognitive flexibility and consequently the occurrence of perseverative errors. The $\phi$ parameter (forgetting speed) negatively correlated with CC ($r = - 0.34$) and showed a moderate positive correlation ($r = +0.40$) with all types of errors with the exception of FMS with which it showed a low but significant correlation. These results suggest that memory decay influences the global cognitive performance of the model with no specificity for errors. The $\tau$ parameter (distractibility) had a similar correlation profile, but showed stronger correlations (mostly above a 0.7 value), in particular a strong positive correlation with FMS ($r = +0.86$). This confirms the important effect that distractibility has on FMS errors. The $\lambda$ parameter (language contribution) showed a positive correlation with CC ($r = +0.32$), and a moderate negative correlation with all errors with the exception of FMS (non-significant correlation). This suggests that inner speech contributes to global performance similarly to the other processes of attention and working-memory. A further analysis of the effects of the parameters on the shape of the landscape of behavioural indices confirms these correlations (Supplementary Materials, Figs. S2–S5).

We carried out an analysis of the simulations where the role of language was negligible ($\lambda < 0.05$; sample size: $n = 175$; here all correlations between $\lambda$ and the behavioural indices became non statistically significant). Table 3 shows the resulting correlations. This analysis highlights the contribution of inner speech to different processes. Indeed, if the contribution of language is strongly reduced and the correlation of the different parameters increases, this means that language can assume a vicarious role with respect to the processes corresponding to those parameters.

The correlation coefficients between $\mu$ (error sensitivity) and most behavioural indices became stronger, in particular with PE (from $r = - 0.09$ to $r = - 0.22$). This indicates that with a weaker language contribution a lower sensitivity to errors increases the model rigidity (perserverance errors); this highlights the important role of language in strengthening the effect of feedback. The positive correlation with FMS ($r = 0.17$) reached significance. This means that $\mu$ can polarise the relevance of the different rules and sharpen their selection, and thus reduce the possibility of mistakenly changing the correct rule: this result indicates that language can play an analogous function. The parameter $\phi$ (forgetting speed) showed a higher correlation with all behavioural indices and in particular the correlation with NPE became stronger (from $r = + 0.40$ to $r = + 0.72$). The stronger effect of forgetting on NPE means that language can play a compensatory role by biasing the activation of specific rules in working memory. The correlation between $\phi$ and FMS, which originally had a negligible statistical significance ($r = + 0.04$, $p < 0.05$), became stronger and statistically more significant ($r = - 0.30$, $p < 0.001$). The implication of this is again that language can strengthen the bias of selecting the correct rule. Conversely, the correlations between $\tau$ (distractibility) and behavioural indices passed from strong to moderate values, in particular the correlation with FMS decreased (from $r = + 0.86$ to $r = + 0.34$). The cause of this, less clear, is possibly that with a reduced role of language the processes associated with $\mu$ and $\phi$ can more strongly contribute to generate the different errors and so distractability becomes less important. A further analysis of the effects of the parameters on the landscape of behavioural indices when language plays a negligible role (Supplementary Materials, Figs. S6–S8), confirms the strengthening and vicarious role of language with respect to other cognitive processes and highlights the presence of relevant linear relations between the $\mu$, $\phi$, $\tau$ parameters and the behavioural indices.

**Table 3:** Pearson’s correlations between key parameters ($\mu$, $\phi$, $\tau$, $\lambda$) and WCST indices in the case of a low language contribution ($\lambda < 0.05$).

Indices	Parameters			
	${\mu }$	${\phi }$	${\tau }$	${\lambda }$
**CC**	0.07	$-$ ***0.63***	$-$ ***0.68***	0.05
**TE**	$-$ 0.10	***0.70***	***0.60***	$-$ 0.09
**PE**	$-$ *0.22*	***0.63***	***0.52***	$-$ 0.12
**NPE**	$-$ 0.03	***0.72***	***0.63***	$-$ 0.08
**FMS**	*0.17*	***0.30***	***0.34***	$-$ 0.04

**Bold** indicates correlations above |0.3| and *Italics* the statistically significant ones ($p<0.05$).

#### Comparison between the behaviour of the model and of human groups

**Figure 3:**
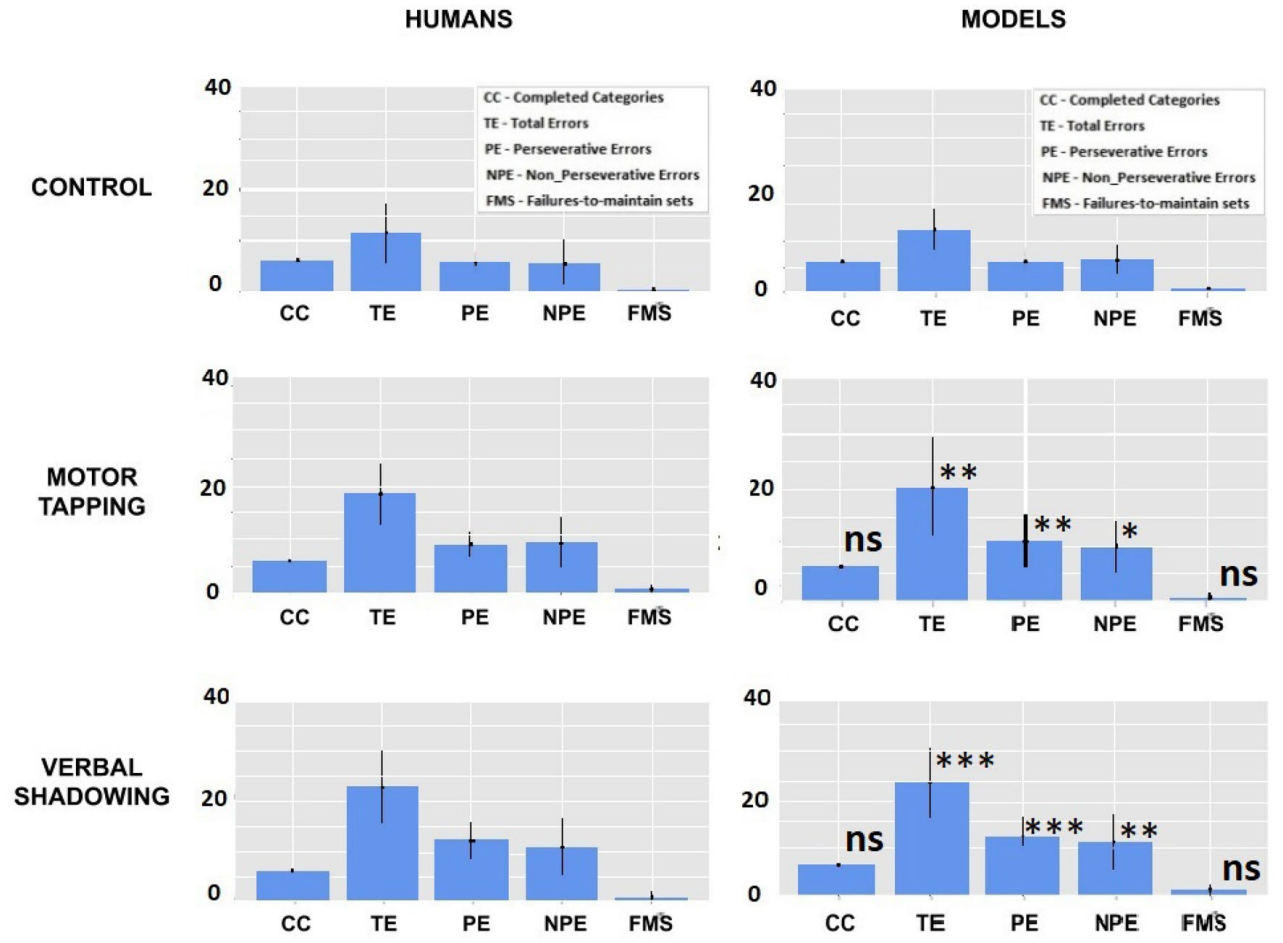
Comparison between human groups (left graphs) and models (right graphs) in the three conditions (rows of graphs) for each behavioural index. The significance asterisks in the model graphs are related to the comparison between each of the motor tapping and verbal shadowing models with the control model: ns = non statistically significant, $p > 0.05$; * = $p < 0.05$; ** = $p < 0.01$; *** = $p < 0.001$.

Figure 3 allows a comparison of the behavioural indexes of the different versions of the model with the indexes of the target human groups (control, motor tapping, and verbal shadowing conditions; see Table S1, Supplementary Materials). The comparison shows that there is no statistical difference between them (p-values of t-tests, double tail, $p>0.05$), thus indicating that the model is very effective in reproducing the behaviour of all the human groups.

Since the model had such a good fit, the role played by the different cognitive processes within the model, quantified by the size of the respective parameters, should reflect an analogous role played in the real participants. Analogously, the *differences* between the different model versions, fitting the behavioural indexes of the real participants in the different conditions, should reveal the different weight of the cognitive processes in their solution of the WCST.

Figure 3 and Table S2, Supplementary Material, show a comparison of the behavioural indexes between the different versions of the model (control model, motor-tapping model, and verbal-shadowing model). While all models did not substantially differ in terms of CC and FMS, the error indexes were higher in the motor tapping model, and even higher in the verbal shadowing model, with respect to the control model. In particular, the motor tapping model exhibited a higher number of total errors with respect to the control group ($18.29 \pm 5.96$ vs. $12.35 \pm 4.14$; $p<0.01$), with a balanced profile of PE/NPE errors. The verbal shadowing group showed an even higher number of total errors ($22.88 \pm 7.34$ vs. $12.35 \pm 4.14$; $P<0.001$), with an analogous balance of PE/NPE. These results indicate that both motor tapping and verbal shadowing cause a general decrease in performance (stronger for verbal shadowing) due to both an increased perseverative behaviour (higher PE) and attentional failure (higher NPE).

### Lesions

This section presents the study of the effects of seven possible alterations (‘lesions’) of the parameters of the control model. Table 4 shows the specific parameters used to produce each lesioned model. Figure S9 in Supplementary Materials shows a graphical overview of the parts of the model architecture that were affected by the lesions. Each of the first three lesions involved a critical parameter regulating a main process of the model, namely error sensitivity, forgetting speed, and distractability. These lesions were supposed to lead to respectively an ‘extreme perseverative model’ (EPM), a ‘distracted model’ (DM), and an ‘irrational model' (IM). We did these lesions to study the compensatory role of language with respect to the functions involved by the parameters. The last four lesions involved the verbal component (inner speech). In particular, the first verbal-lesion model (VLM1) involved a reduced contribution of language in case of external negative feedback. The second verbal-lesion model (VLM2) involved a reduced contribution of language in case of external positive feedback. The third verbal-lesion model (VLM3) involved a low language contribution during the storing of working memory. The fourth verbal-lesion model (VLMG) involved a global lesion including all previous verbal lesions. Table S3, in Supplementary Materials, shows a statistical comparison of the behavioural indices between the control model and the lesioned models.

**Table 4:** Parameters of the lesioned models obtained by altering the parameters of the control model that fits the human control group (data reported in^47^).

	$\mu$	$\phi$	$\tau$	$\lambda$
Control model	0.49	0.97	0.10	0.81
Extreme perseverative model (EPM)	***0.001***	0.97	0.10	0.81
Distracted model (DM)	0.49	0.97	***0.4***	0.81
Irrational model (IM)	0.49	***1.0***	0.10	0.81
First verbal-lesion model (VLM1)	0.49	0.97	0.10	***0.001*** (only if r = 0)
Second verbal-lesion model (VLM2)	0.49	0.97	0.10	***0.001*** (only if r = 1)
Third verbal-lesion model (VLM3)	0.49	0.97	0.10	***0.001***(r not considered)
Global verbal-lesion model (VLMG)	0.49	0.97	0.10	***0.001***

The first three models involve lesions of the main cognitive processes of the model, while the last four models involve four different lesions of the language component. Values in ***bold Italics*** represent the parameters that were altered to produce the lesioned models.

Both the EPM and IM do not show any statistical difference in the behavioural indices compared to the control model. This result suggests that high functioning language component ($\lambda = 0.81$ in both cases) compensates low error sensitivity (EPM has $\mu = 0.001$) and high forgetting (IM has $\phi = 1.0$), highlighting the vicarious role that language can play. Conversely, the DM shows a statistically significant difference for each index ($p < 0.001$ for each index). In particular, it shows a lower CC ($0.88 \pm 0.9$ vs $6.0 \pm 0$), a higher TE ($47.65 \pm 6.39$ vs $12.35 \pm 4.14$), formed by 40% of PE and 60% of NPE, and a higher FMS ($2.71 \pm 1.7$ vs $0.59 \pm 0.69$). This is coherent with the theoretical interpretation of the $\tau$ parameter representing distractibility since high distractability cannot be compensated for by language.

The VLM1 (impairment of the language contribution in case of negative feedback) showed a statistical significant difference with each index of the control model, with the exception of CC and FMS. In particular, the model showed a higher TE ($20.41 \pm 8.35$ vs $12.35 \pm 4.14$, $p < 0.01$), composed by 48% of PE and 52% of NPE. Overall, this lesioned model did not show a great global impairment, as shown by the high CC and a slightly higher number of total errors. The reason is that in this model the impairment only corrupts the language contribution in case of negative feedback and this is compensated by an intact high error-sensitivity coefficient ($\mu = 0.49$).

The VLM2 (impairment of the language contribution in case of positive feedback) showed worse performances compared to both the INVM and control models. In particular, it showed a very low CC ($0.06 \pm 0.24$ vs $6.0 \pm 0$, $p < 0.001$) and a very high TE ($54.71 \pm 4.08$ vs $12.35 \pm 4.14$, $p < 0.001$) formed by 35% of PE and 65% of NPE. With the exception of FMS, that is not statistically different from the one of the control model, the error profiles of this model are similar to those of the DS model ($\tau$ = 0.4), showing a high number of errors and in particular an imbalance toward NPE, thus suggesting an attention impairment. This suggests that language plays an ‘attentional focus’ function that in case of positive feedback increases the probability of focusing on the specific correct rule discovered and stored in memory.

The model VLM3 (impairment of the language contribution to the storing function based on the phonological-loop) did not show any statistical difference in any behavioural index compared to the control model. This suggests that the simple storing function of inner speech has not a relevant role in this task.

The VLMG (global impairment of the language contribution) exhibits the worst indexes with respect to all models. In particular, the model has a very low CC ($0.12 \pm 0.32$ vs $6.0 \pm 0$, $p < 0.001$) and the highest TE ($60.82 \pm 4.66$ vs $12.35 \pm 4.14$, $p < 0.001$), formed by 35 % of PE and 65% of NPE, and a higher FMS ($1.24 \pm 0.94$ vs $0.59 \pm 0.69$, $p< 0.05$). The error profile of this model is similar to the one of the DM but shows worse indices (with the exception of FMS). This result suggests that a global impairment of the language system causes a severe deterioration of the model flexible goal-directed behaviour.

### Internal functioning of the model

**Figure 4:**
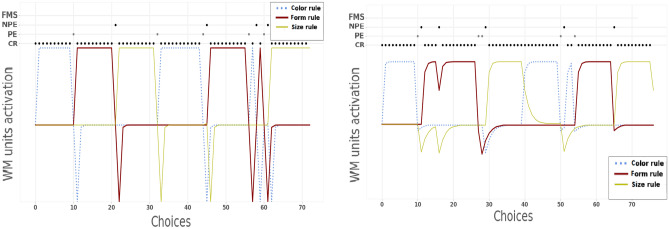
Internal functioning of two control models. Left: model with language. Right: model without language. Each line in the graphs shows the activation of a working-memory unit representing a tendency to choose a specific sorting rule between the three possible rules. The dots at the top of graphs indicate single instances of correct responses (CR) or errors (PE, NPE, FMS).

Here we show the internal functioning of the model mimicking the control group (Fig. 4, left) and the three models each with a specific lesion of the inner-speech component (VLM1, VLM2, VLMG; see Fig. S10 in Supplementary Materials). We do not consider the model with an impaired phonological loop function (VLM3) because it did not show any statistically relevant difference with the control model. We also show a plot related to a model without the language system and fitting the data of the human control group (Fig. 4, right). This model shows what happens if one assumes that during development the absence of the inner-speech component would be substituted by vicarious cognitive processes still supporting flexible behaviour. Note that the different models might also capture individual differences in the use of inner-speech as a support for high-level cognitive processes, as highlighted in^47^. The two control models have a similar good performance (they both solve the task in 80 rounds) but they exhibit a partially different internal functioning that highlights the role that language can play in the solution of the task (Fig. 4).

Notwithstanding the similar behaviour of the two models, the qualitative comparison of the activation of the working-memory units encoding the different decision rules shows that they are more ‘disentangled’ in the model with the language component. In particular, in the model with language the units have a more polarised activation and sharper activation changes. This supports a higher cognitive flexibility, in particular rapid decreases of activation after an error, and a high focused capacity, in particular rapid increases of activation after a positive feedback.

Figure S10, in Supplementary Materials, shows the internal functioning of the three verbal-lesion models. The VLM1 (language impairment in case of negative feedback) has an internal functioning similar to the one of the control model, but also some differences. In particular, the high information forgetting speed ($\phi = 0.97$, shared with the control model) causes a fast decay of the activation to 0.5 (baseline activation of units) while the absence of a linguistic contribution in case of negative feedback prevents strong decrements of the activation of units. The effect of these two specific processes causes a minor difference between the activation of the units (in particular an higher inferior boundary) thus producing more PE (e.g., see choice interval 28–31) and NPE (e.g., see choice interval 70–75).

The VLM2 (language impairment in case of positive feedback) shows an erratic and inefficient internal functioning. In particular, it shows a lower superior bound of activation and sudden extreme activation changes that do not allow the completion of the test and produce several PE and NPE. Paradoxically, the erratic behaviour causes also random completions of categories (e.g., see choice interval 35–44). This result shows that the language contribution after a positive feedback supports the focus on a specific rule for prolonged times.

The VLMG (all language impairments) showed an internal behaviour similar to an average behaviour of the previous two models. In particular, it exhibits a minor range of activation (inferior and superior bounds) and erratic changes of activities that prevent the completion of any category. Interestingly, this plot is qualitatively more similar to the one of VLM2 (positive feedback and focusing impairment) than to the one of VLM1 (negative feedback impairment), thus corroborating the previously discussed quantitative data indicating that the language contribution to focusing is more important than its contribution to processing feedback errors.

## Discussion

The model proposed here highlights the specific mechanisms through which inner speech might enhance the internal manipulation of representations involved in goal-directed cognitive processes and executive functions. In particular, it accounts for the cognitive flexibility as measured in the Wisconsin Card Sorting Test (WCST).

Theory-driven statistical analyses, focusing on versions of the model having parameters involving a negligible role of language, highlighted the similarity of the functions played by feedback-based working-memory update processes, and those played by language processes, both capable of supporting the *attentional focusing on successful goals* (behavioural rules). In addition, the comparison between the control model and versions of the model whose language *storing function* was lesioned did not show significant statistical differences, thus suggesting that the support of language for this function has a negligible role in the target experimental test. These results corroborate the importance of working-memory in flexible cognition as measured in the WCST, highlighting its role of ‘executive function’ rather than as mere information storage^63^. The results presented here thus suggest that inner speech can play a key role in enhancing the capacity of manipulating the states of working memory and thus to improve the effectiveness of goal-directed behaviour.

We also analysed the internal functioning and interaction of the system components. This analysis involves the control model with and without language and the language-lesioned versions of the model. Results indicate that the role of inner speech to enhance the executive-function role of working memory is based on its capacity to strengthen the activation differences between neural units which represent alternative possible goals (the behavioural rules to follow in the solution of the WCST). Once so differentiated, the internal representation carrying relevant information are more robust to lesions, distractions, and internal/external sources of noise. This ‘disentanglement’ function of language also manifested in a previous abstract non-embodied computational model^64^ further discussed in section "Comparison with other models of language–cognition interaction". Interestingly, artificial intelligence has recently started to highlight and study the importance for neural-network architectures to be able to ‘disentangle’ internal representations to enhance the signal/noise ratio for downstream processing components^65,66^.

**Figure 5:**
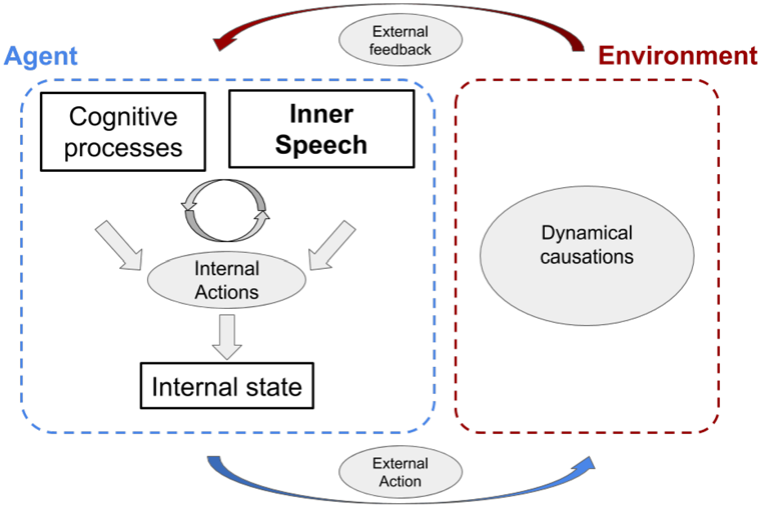
Abstract schema that represents the model double loop of manipulation of internal and external states. The internal manipulation of states allows the agent to better manipulate the external environment.

Overall, these results point to a possible *super-ordinate role* of inner speech that involves both executive functions and perceptual embodied processes. In this perspective, inner speech represents a boosting internal cognitive ‘tool’, as highlighted by the psychological literature discussed in section “Introduction”. This view leads us to suggest the existence of two goal-directed embodied-manipulation loops supported by inner speech (Fig. 5). The first loop involves the classic embodied interaction of the agent with external objects, e.g. the visual search and the card manipulation processes performed to solve the WCST. The second loop involves inner speech in its role as cognitive tool, in particular to enhance the manipulation of internal representations stored in working memory and so improve the effectiveness of the first loop.

### Comparison with other models of the language–cognition interaction

Few scientific studies focus on the interaction between language and cognitive processes adopting a theoretical and computational approach. Here we review computational models that contribute to investigate this important topic.

A first biologically grounded model simulates the brain networks supporting the interaction between attention and language^67^. This model has high biological fidelity, simulating the neurophysiologically and anatomically grounded networks supporting the perception and production of speech. The model uses winner-take-all neural mechanisms to reproduce bottom-up attentional selection of words and non-words at different brain levels. However, the model does not investigate how goal-based top-down processes might affect internal representations. Another computionally more sophisticated model^68^, expanding the previous model, simulates the brain neural networks supporting decision-making processes for action and speech. The model is used to explain the spontaneous emergence of intentional speech acts in the brain. The model has the same limitations of the previous model and in addition investigates the influence of high-order processes on language but not the influence of language on cognition as done here.

More abstract models investigate the interaction between language and cognition in particular focusing on the supporting role played by language for categorisation processes. A first model^69^ links symbolic processing (words) to neural distributed representations and implements deep neural-network architectures involving sensory-motor learning and symbolic learning. The model investigates the top-down effect of symbolic computations on neural-network representations, suggesting that language can represent a ‘symbolic theft’ tool to improve categorisation. This model is not validated with empirical data as here and addresses a different investigation problem with respect to the role of language for the internal manipulation of representations.

Three further models show mechanisms that use ‘linguistic labels’ to influence the computations of neural networks. The first model^70^ is based on an auto-encoder neural-network. The model is used to show how the injection of linguistic labels into the intermediate layers of the model can enhance its classification capabilities. The model has an abstract architecture that cannot be mapped onto specific cognitive processes and is not validated with empirical data, but it nevertheless proposes an interesting mechanism for which language can manipulate the internal representations of auto-encoder neural networks. Another computational model^64^ highlights a possible role of inner speech for cognition. The model in particular includes two simple neural networks that model a sensory-motor loop, learning to categorise objects, and a phonological loop, learning to repeat words. The two networks interact at the level of their intermediate layers. Although the simple architecture does not capture specific cognitive processes and the model is not validated with empirical data, the results show that self-directed language can enhance the disentanglement of internal representations for different categories of objects, a phenomenon also emerged here. A last model^7^ solves a sensory-motor classification tasks and shows how language can be used with vicarious functions with respect to visual inputs and to activate goals allowing to flexibly respond to stimuli. The model is qualitatively validated with empirical data but it does not investigate how language might influence the manipulation of internal representations.

Overall, compared to all aforementioned models the model presented here has an architecture directly capturing key high-level cognition processes. Moreover, it allows the study of how language can act as a cognitive tool supporting the manipulation of internal representations enhancing the interaction with the external environment. In so doing, it focuses on a superordinate role of language that can potentially explain its influence on many different domain-specific tasks (e.g., object categorisation or cognitive flexibility). Due to these differences, the architecture represents a more accurate model of self-directed language for executive functions.

## Conclusions

This work investigates the interaction between self-directed language (inner speech) and goal-directed cognition through a computational model. To our knowledge, the model represents the first integration of inner-speech mechanisms within a cognitive architecture capable of manipulating internal representations that support goal-directed behaviour. The model reproduces human behavioural data during the performance of the Wisconsin Card Sorting Test, designed to study cognitive flexibility, both in a standard condition and in a condition involving verbal shadowing.

The model allowed us to show how language can play key functions to support the cognitive processes, nemely the processing of external feedback and working memory. In particular, the model suggests that inner speech ameliorates attention engagement and disengagement with respect to specific goal representations after a feedback is received, and in general augments the disentanglement of goal representations. This ‘sharpens’ cognitive processes, making them more robust to lesions, distractors, and noise. Overall, the model reveals how self-directed language can strengthen the effectiveness of various cognitive process involving the manipulation of internal representations and thus improve cognitive flexibility and the interaction with the environment.

## Supplementary information

Supplementary material 1
