# Supplementary material for: A computational model of language functions in flexible goal-directed behaviour
Source: Sci Rep. 2020 Dec 10;10:21623. doi: 10.1038/s41598-020-78252-y (PMC7729881; doi:10.1038/s41598-020-78252-y)
Supplement: Supplementary file 1 — Supplementary material 1 [file 41598_2020_78252_MOESM1_ESM.pdf]

# A COMPUTATIONAL MODEL OF LANGUAGE FUNCTIONS IN FLEXIBLE GOAL-DIRECTED BEHAVIOUR *SUPPLEMENTARY MATERIALS*


**Giovanni Granato**
Laboratory of Computational Embodied Neuroscience
Institute of Cognitive Sciences and Technologies
National Research Council of Italy,
Rome, Italy
giovanni.granato@istc.cnr.it

Anna M. Borghi
Department of Dynamic and Clinical Psychology
Sapienza University of Rome
Institute of Cognitive Sciences and Technologies
National Research Council of Italy
Rome, Italy
anna.borghi@uniroma1.it

Gianluca Baldassarre*
Laboratory of Computational Embodied Neuroscience
Institute of Cognitive Sciences and Technologies
National Research Council of Italy
Rome, Italy
gianluca.baldassarre@istc.cnr.it


July 30, 2020


## ABSTRACT

The function of language in high-order goal-directed human cognition is an important topic at the centre of current debates. Experimental evidence shows that inner speech, representing a self-directed form of language, empowers cognitive processes such as working memory, perception, categorization, and executive functions. Here we study the relations between inner speech and processes like feedback processing and cognitive flexibility. To this aim we propose a computational model that controls an artificial agent who uses inner speech to internally manipulate its representations. The agent is able to reproduce human behavioural data collected during the solution of the Wisconsin Card Sorting test, a neuropsychological test measuring cognitive flexibility, also when a verbal shadowing protocol is used. The components of the model were systematically lesioned to clarify the specific impact of inner speech on the agent's behaviour. The results indicate that inner speech improves the efficiency of internal manipulation. Specifically, it makes the representations linked to specific visual features more disentangled, thus improving the agent's capacity to engage/disengage attention on stimulus features after positive/negative action outcomes. Overall, the model shows how inner speech could improve goal-directed internal manipulation of representations and enhance behavioural flexibility.

## Parameters estimation: details

The parameters of simulations that best represents the three experimental conditions studied here (control, motor tapping and verbal shadowing) were searched through a brute-force algorithm [1]. In particular, the algorithm searched the values of the parameters that minimise the difference between the WCST indices obtained by the model and those found in humans. To apply the algorithm, we randomly-sampled $3,000$ combinations of parameters each drawn with a uniform distribution in the following ranges: $\phi$: $(0.0, 1.0)$; $\mu$: $(0.0, 1.0)$; $\tau$: $(0.0, 0.3)$; $\lambda$: $(0.0, 1.0)$. For each parameter combination, we then performed 30 simulations of the task, so obtaining an average value of the WCST indices. For each parameter combination, these average values were then compared with the average values of the target human groups reported in [2]. This comparison was in particular based on the *Mean Squared Error* (MSE) computed as follows:

$$MSE = \frac{||\mathbf{y} - \mathbf{y}'||_2^2}{n} \tag{1}$$

where $\mathbf{y}$ is the vector of mean indices of the human group, $\mathbf{y}'$ is the vector of mean indices of the considered parameter combination, $|| \cdot ||_2^2$ is the square of the L2 norm, and $n$ is the length of vectors.

## Computational details of the model

**Environment** The simulated environment component of the simulations is composed by the objects (cards) which the model can visually explore (visual search) and on which it can executes a physical action (displacement). The cards are polygons with a unique combination of three visual dimensions (colour, form, and size), each having one of four possible attributes: colour (red, green, blue, yellow); form (square, circle, triangle, bar); size (large, medium-large, medium-small, small). There are thus $4^3 = 64$ combinations (cards) of attributes.

**Visual sensor** The visual sensor covers a limited portion of the whole environment, returning a $28 \times 28 \times 3$ RGBY pixel matrix. This matrix is unrolled and stored in a vector of 2352 elements representing the perceptual input to the model. The visual sensor is actively moved in the scene under the guidance of a top-down visual search process that produces object-centred sensor movements (saccades), first directed toward the deck and then sequentially toward the target cards.

**Working-memory** The working-memory is formed by three recurrent units (each unit has a self-connection) which can acquire a continuous value ranging in $[0, 1]$. The activation of each unit represents the likelihood of selection that the system assigns to each of the three possible matching rules of the task related to colour, form, and size. The activation of the each unit is characterised by an internal decay toward a baseline (0.5) and is described by the following equation:

$$m_{l,t} = (1 - \phi) \cdot m_{l,t-1} + \phi \cdot \alpha = m_{l,t-1} + \phi(-m_{l,t-1} + \alpha) \tag{2}$$

where $m_{l,t}$ is the value related to a losing unit $l$ ($l \in 1, 2, 3;\ l \neq s$, where $s$ is the selected unit considered below) at time $t$, $1 - \phi$ is the strength of the recurrent connection, and $\alpha = 0.5$ is the baseline value to which the memory unit activation converges. The parameters $\phi$ is a critical parameter of the model investigated in the simulations. A high value of this parameter causes a high rate of information forgetting.

**Motivational component** This component is supported by a reinforcement algorithm. In particular it receives the external feedback signal (a binary value in $\{0, 1\}$) and subsequently affects the activation of the unit encoding the last selected and used rule. This process is described as follows:

$$m_{s,t} = (1 - \mu) \cdot m_{s,t-1} + \mu \cdot r = m_{s,t-1} + \mu(-m_{s,t-1} + r) \tag{3}$$

where $m_{s,t}$ is the new activation of the rule unit, $s \in \{1, 2, 3\}$ is the index of the selected rule, $m_{s,t-1}$ is the current activation of the unit, $(1 - \mu)$ is the strength of the unit recurrent connection, $\mu$ regulates the impact of the feedback on the memory, and $r$ is the feedback signal that is equal to 1 in case of positive feedback (correct matching of the deck card and target card) and 0 otherwise. The parameter $\mu$ is set to a fixed value of 0.7 for positive feedback and to a variable value for the negative feedback. The latter value plays a critical role for the model and is investigated in the simulations.

**Hierarchical perceptual component** This component is supported by a deep generative model, in particular a *Deep Belief Network* (DBN, [3]) composed of two stacked *Restricted Boltzmann Machines* (RBM). A RBM is a bidirectional-graph model formed by two layers of fully-connected units. We trained the first RBM (composing the input layer and the first hidden layer of DBN) with a standard learning rule for this model (*contrasting divergence*, [4]). This

algorithm learns the joint probability of hidden layer and input layer activations. This allows the network to 'reconstruct' ('generate') the original input trough a bidirectional activation from the input layer, to the hidden layer, and then back to the input layer. We trained the second RBM (composed by the first and second hidden layers of the DBN) with a modified version of the original algorithm that allows us to alter the reconstructions of original inputs to obtain prototypical representations of input image features on which the system focuses on (e.g., in case of a focus on colour, a red triangle given as input is reconstructed as a shapeless red blob). This modification causes the emergence of three groups of units in the last layer of DBN (its second hidden layer), each corresponding to specific visual categories of the input (first four units for colour: red, green, blue, yellow; second four units: square, circle, bar, triangle; third four units: small, medium-small, medium-large, large). The reconstruction images produced by an inverse activation from the last hidden layer of DBN to the input layer constitute the input for the comparator component. In particular, the selector and manipulator considered below are able to select one category (one group of four units), and one attribute within it (one neural unit), to produce the prototypical rule-based reconstruction of images mentioned above.

**Selector and manipulator components** The selector is represented by a winner-take-all (WTA) competition that receives the values from the working memory as input, and chooses the matching rule on the basis of a $softmax$ function:

$$Pr(k = s) = \frac{\exp(m_k \,/\, \tau)}{\sum_{q=1}^{3} \exp(m_q \,/\, \tau)} \tag{4}$$

where $Pr(k = s)$ is the probability that the matching rule $k$ ($k \in 1, 2, 3$) is selected ($k = s$). The parameter $\tau$ of the $softmax$ function, called 'temperature', regulates the randomness of the selection and is the third important parameter manipulated in the simulations. A high value of $\tau$ causes a high randomness/exploration of the decisions. The probabilities $Pr(\cdot)$, summing up to 1, are used to stochastically select the matching rule to use. The manipulator leads the winner unit of the WTA competition to disinhibit, through a double inhibition mechanism, the units of a specific group corresponding to the chosen category, within the last layer of the perceptual component. In particular the manipulator is composed by two layers of 3 units, linked with one by one negative projections. Each unit of the second layer is always active and has negative projections to a specific group of the last layer of the perceptual component, so the activation of a specific unit in the first layer of the manipulator causes a disinhibition of the corresponding group of the last layer of the perceptual component. Moreover, the manipulator implements a *Hard-max* function leading to select only one unit (attribute) within the each group (category) of four units.

**Verbal component** This component is formed by a multi-layer perceptron (MLP). This component receives one-to-one connections from the selector units and sends one-to-one connections to the WM units. This process is in particular implemented as follows:

$$m_t = m_{t-1} + \lambda \cdot L_t \tag{5}$$

where $m_t$ is the new activation of a WM rule unit, $m_{t-1}$ is the current activation of the WM unit, $\lambda$ represents the strengths of the one-to-one connection weights linking the language component output-layer units to the WM units, $L_t$ is the current activation of the language component output layer caused by the previous selector units' activation (this time mismatch implies that the component implements a phonological memory). The MLP architecture is formed by $4$ input units, 10 sigmoid hidden units, and 3 output linear units.

The input-layer $4$ units encode: (a) the selector winner-takes-all *one-hot vector* activation; (b) the binary incorrect/correct match feedback encoded with respectively 0/1. The MLP was trained to activate the output-layer 3 units as follow: the unit corresponding to the selected rule learned to produce a $-1/+1$ value based on the match/mismatch feedback; the other two units activated with $0$. For example, if the model chooses the colour rule and receives a positive feedback, the input is $[1, 0, 0, 1]$ and the desired output is $[1, 0, 0]$; conversely, if the model chooses the colour rule and receives a negative feedback the input is $[1, 0, 0, 0]$ and the desired output is $[-1, 0, 0]$. The language component is activated two times to simulate: (a) the phonological-loop working memory; (b) the feedback-dependent verbal update of the main working memory. In the first activation, the component input layer is activated by the one-hot code of the selector while its feedback unit is activated with $1$ (meaning 'maintenance of the current rule'). In the second activation, the component input layer is activated by the selector activation, but in this case the feedback unit value is activated on the basis of the external feedback (0/1), obtained after the action execution (displacement of the card). The contribution of language to the working memory is regulated by a coefficient $\lambda$ that ranges in $[0, 1]$ and represents the strengths of the one-to-one connection weights linking the language component output layer to the main working memory units. The coefficient $\lambda$ is the fourth and last important parameter regulating the functioning of the model and investigated in the simulations. The language MLP component is trained before the experiments illustrated in the main text with a supervised learning algorithm [5]. In particular, the system is trained with six different input patterns and six different corresponding output patterns encoding respectively the three possible rules and the binary valence with which to activate the units of the main working memory. The learning rate was set to $0.01$ and the network was trained till convergence.

**Visual comparator** This component executes a mathematical comparison (Euclidean distance) between the two reconstructed images corresponding to the deck card and the currently-foveated target card returned by the perceptual component. The component includes a fixed threshold ($\beta = 0.1$) and returns a Boolean value representing the result of the comparison ('same'/'not same').

**Motor component** This component simulates two mechanisms allowing [6]: (a) the top-down visual search, i.e.. the saccades corresponding to the displacement of visuals sensor, and (b) the interaction of the model with environment, in particular the displacement of the deck card from the deck to specific target card. The first mechanism receives the position (Cartesian coordinates) of the deck card and the target cards and displaces the visual sensor on them in a sequential manner. The second mechanism receives the position of the deck card and of the matched target card (Cartesian coordinates) and displaces the deck card toward the position of the matched target card.

## Results

### Statistical analysis of the relation between parameters and behavioural indices

Figure S1 shows the parameter values for the control, motor tapping, and verbal shadowing conditions.

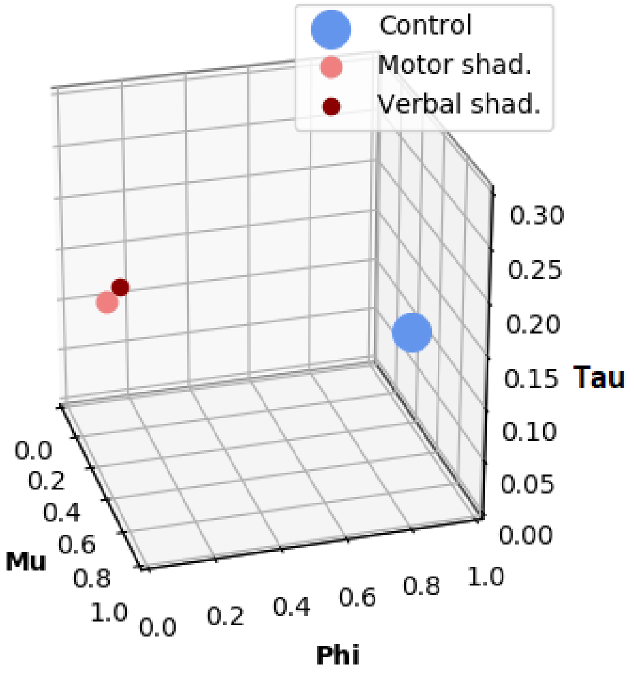


Figure S1: 3D plot of the model parameter configurations that best fit the corresponding human groups for each condition (control, motor tapping, and verbal shadowing). The size of each coloured sphere represents the magnitude of the language contribution (parameter $\lambda$).

To investigate the type of relation between each model parameter and the behavioural indices, we performed the following analysis. For each parameter, we considered all simulations with a parameter combinations where: (a) the parameter under focus could have any value within its range; (b) the other parameters could have a value within a narrow range around the value they had in the the control model; the parameters of the control model used to this purpose had the values:

$\mu = 0.49$, $\phi = 0.97$, $\tau = 0.10$, $\lambda = 0.81$; and the narrow ranges were: $\mu \in (0.4, 0.6)$, $\phi \in (0.7, 1)$, $\tau \in (0.09, 0.12)$, $\lambda \in (0.65, 0.85)$.

The results of this analysis is shown in Figure S2 - Figure S5 related to the effect of each parameter on the five behavioural indices. The results show that: (a) the found relations agree with the statistical correlations reported in the

main text (Pearson $r$, Table 2); (b) show that the parameter-indices relations have a relevant linear component (although there are other non-linear relations captured by correlation indices); (c) the ‘index landscape’ is relatively regular and the parameter-index relation has a relevant linear component.

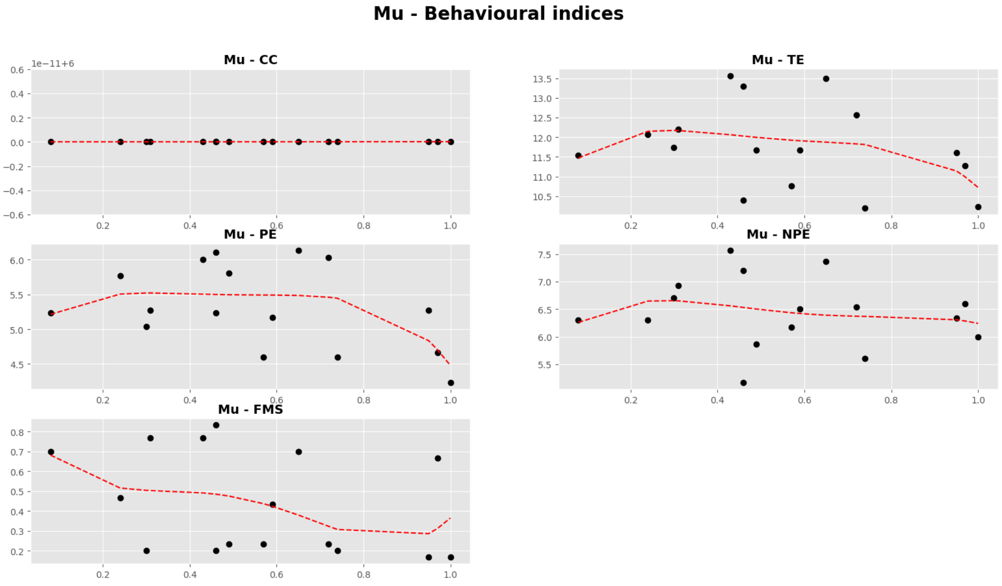


Figure S2: Values (y-axis) of the five behavioural indices (different graphs) corresponding to the different values of the parameter $\mu$ (x-axis). The range of the other parameters was chosen within a range around the values of the control model (see text). Red dashed line: 4-degree polynomial regression.

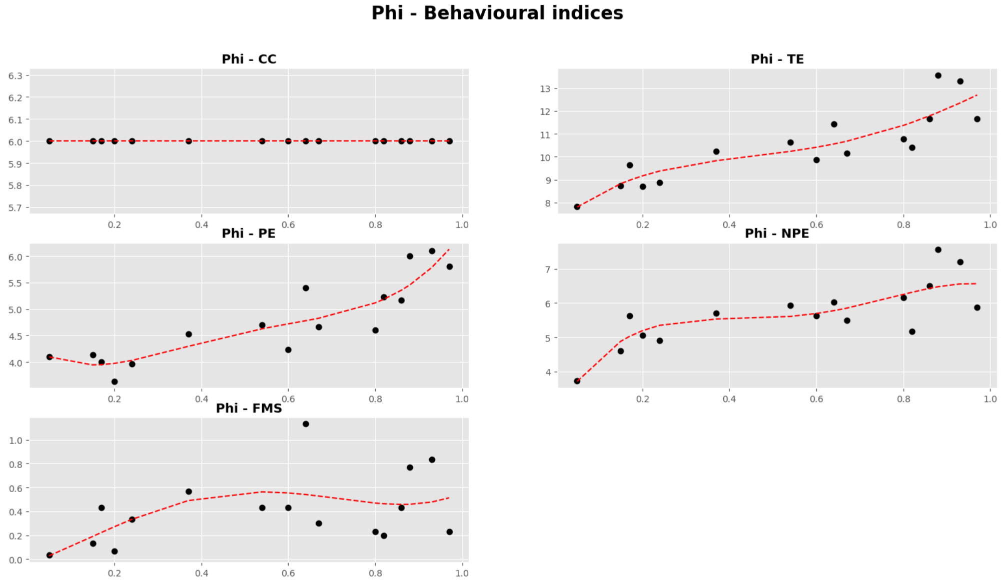


Figure S3: Values (y-axis) of the five behavioural indices (different graphs) corresponding to the different values of the parameter $\phi$ (x-axis). The range of the other parameters was chosen within a range around the values of the control model (see text). Red dashed line: 4-degree polynomial regression.

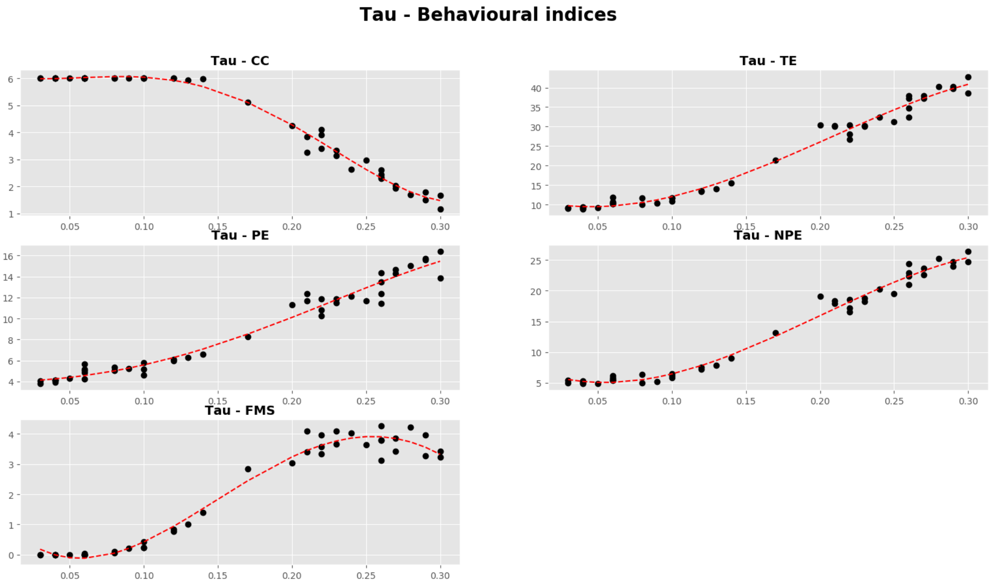


Figure S4: Values (y-axis) of the five behavioural indices (different graphs) corresponding to the different values of the parameter $\tau$ (x-axis). Red dashed line: 4-degree polynomial regression.

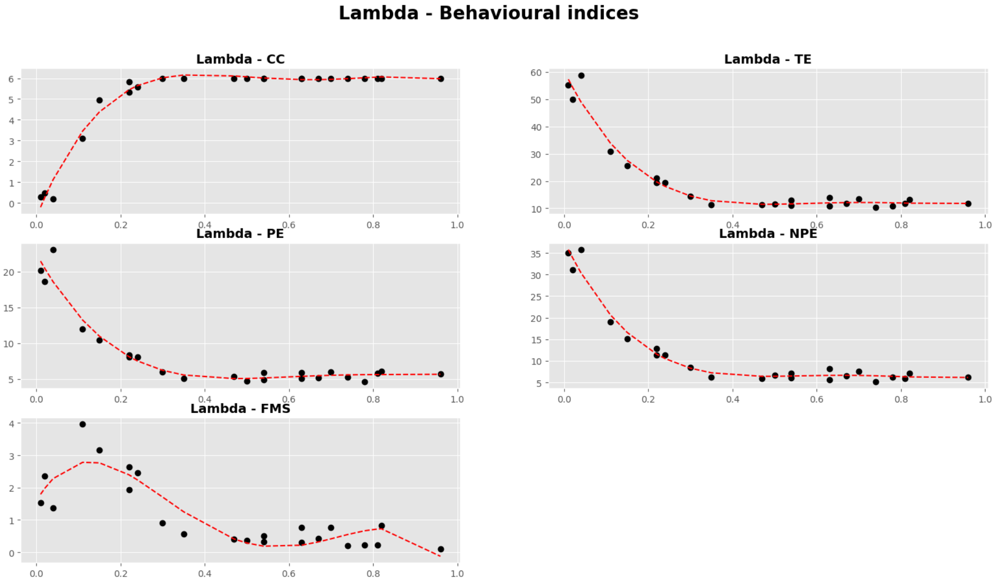


Figure S5: Values (y-axis) of the five behavioural indices (different graphs) corresponding to the different values of the parameter $\lambda$ (x-axis). The range of the other parameters was chosen within a range around the values of the control model (see text). Red dashed line: 4-degree polynomial regression.

We then perform a second analysis using an analogous method to better understand the role of the model error sensitivity($\mu$), forgetting speed ($\phi$), and distractibility ($\tau$) in the case of absence of inner-speech ($\lambda$).In particular, we performed this analysis with: (a)$\lambda < 0.05$ (low-language effect); (b) an 'ideal' range for the other parameters; this 'ideal range' is a range that we previously ascertained to contain the values that guarantee a very good general

performance of the model and few behavioural errors: $\mu \in (0.5, 1)$ (high error sensitivity), $\phi \in (0.0, 0.5)$ (low forgetting), $\tau \in (0.0, 0.15)$ (low distractibility), $\lambda \in (0.0, 0.05)$ (negligible role of language). The results of this further experiment (Figure S6 - Figure S8) show the interaction between the parameters and the behavioural indices in the case of lack of language. For example, without language the parameter $\mu$ (Figure S6) shows a stronger negative correlation with perseverative errors and error sensitivity for the lack of the vicarious role of language. Moreover, the parameter $\phi$ (Figure S7) shows a stronger negative relation with the overall performance of the model (CC), again for the lack of the vicarious role of language. Last, the parameter $\tau$ (Figure S8) confirms its correlations with each index as its function is not much vicariated by language.

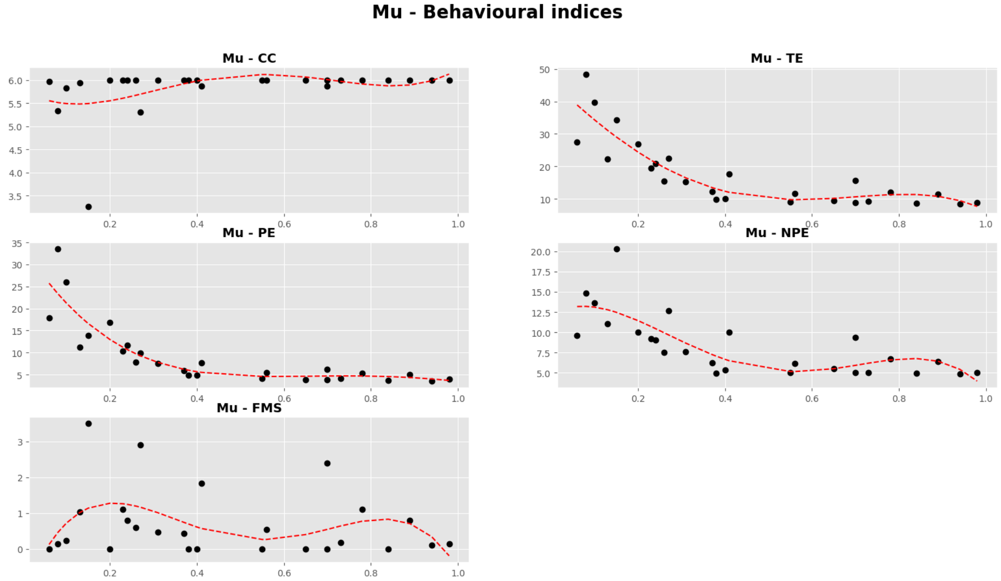


Figure S6: Values (y-axis) of the five behavioural indices (different graphs) corresponding to the different values of the parameter $\mu$ (x-axis). The range of parameter $\lambda$ was chosen within very low values (negligible language) while the other parameters were chosen within ‘ideal values’ (see text). Red dashed line: 4-degree polynomial regression.

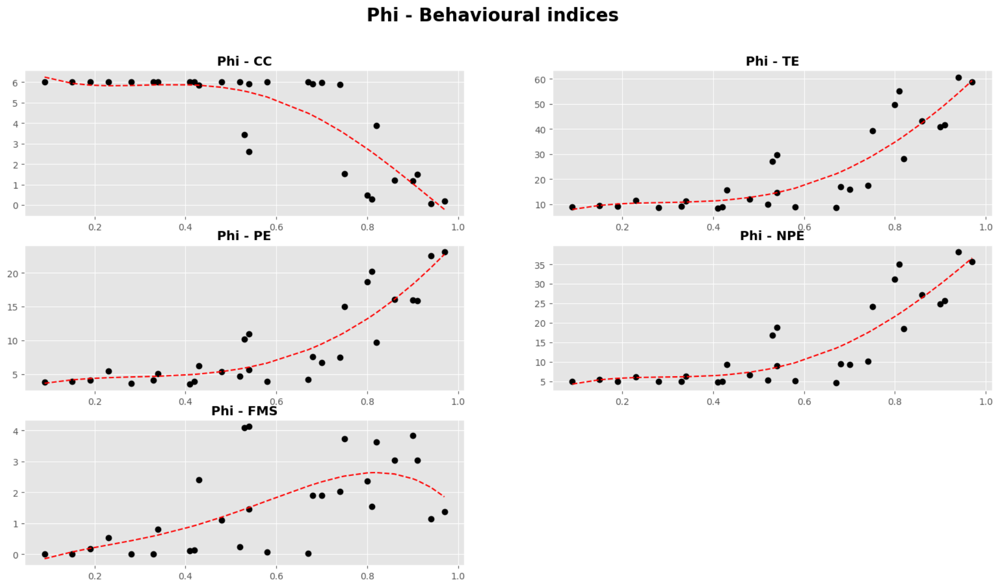


Figure S7: Values (y-axis) of the five behavioural indices (different graphs) corresponding to the different values of the parameter $\phi$ (x-axis). The range of parameter $\lambda$ was chosen within very low values (negligible language) while the other parameters were chosen within ‘ideal values’ (see text). Red dashed line: 4-degree polynomial regression.

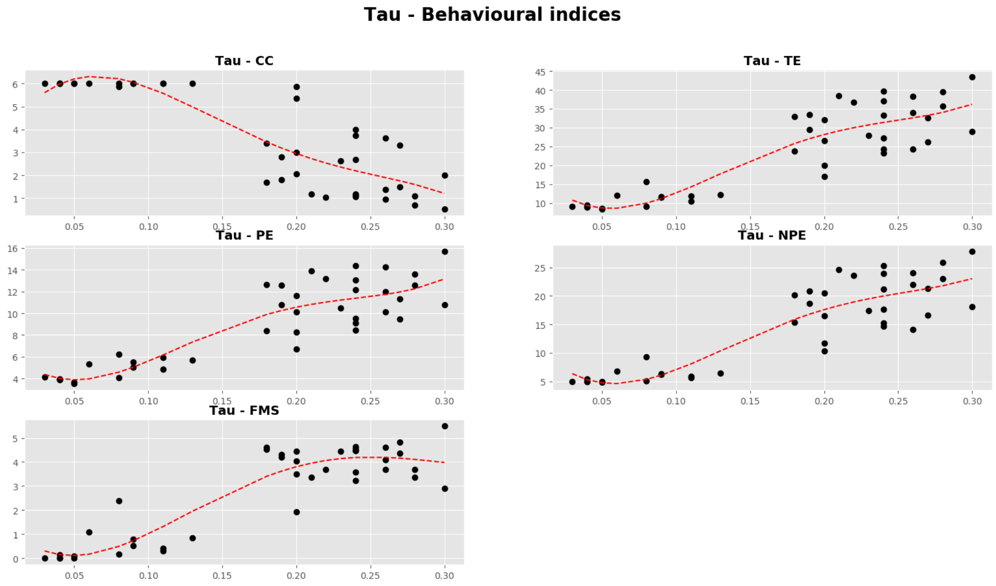


Figure S8: Values (y-axis) of the five behavioural indices (different graphs) corresponding to the different values of the parameter $\tau$ (x-axis). The range of parameter $\lambda$ was chosen within very low values (negligible language) while the other parameters were chosen within ‘ideal values’ (see text). Red dashed line: 4-degree polynomial regression.

### Comparison between the behaviour of the model and of the human groups

Table S1-S2 report the comparison between the model and human data and between different models.

| Participants | Indices | | | | |
|---|---|---|---|---|---|
| | CC | TE | PE | NPE | FMS |
| **Control** | = | .536 | .589 | .571 | 0.102 |
| **Motor tap.** | = | .405 | .222 | .736 | .698 |
| **Verbal shad.** | 0.070 | .380 | .649 | .329 | .989 |

Table S1: Statistical comparisons (p-values, two-tail t-tests) between the behavioural indices of the human groups and the best fitting models, for each protocol condition (control, motor tapping, verbal shadowing; data from [2]). The symbol '=' means an identical value of CC (equal to $6 \pm 0$). All p-values are non statistically significant ($p > 0.05$).

| Models | Indices | | | | |
|---|---|---|---|---|---|
| | CC | TE | PE | NPE | FMS |
| **Control** | $6.0 \pm 0.0$ | $12.35 \pm 4.14$ | $6.06 \pm 1.92$ | $6.29 \pm 2.97$ | $0.59 \pm 0.69$ |
| **Motor tap.** | $6.0. \pm 0.0$ (ns) | $18.29 \pm 5.96$ (**) | $9.06 \pm 2.53$ (**) | $9.24 \pm 4.67$ (*) | $0.59 \pm 0.69$ (ns) |
| **Verbal shad.** | $6.0 \pm 0.0$ (ns) | $22.88 \pm 7.34$ (***) | $12.06 \pm 3.8$ (***) | $10.82 \pm 5.74$ (**) | $0.76 \pm 1.0$ (ns) |

Table S2: Mean and standard deviation of the behavioural indexes of the control, motor-tapping, and verbal-shadowing models. The asterisks indicate the significance (two-tail t-tests) of the statistical difference between the motor-tapping and verbal-shadowing models with respect to the control model. CC = completed categories, TE = total errors, PE = perseverative errors, NPE = non perseverative errors, FMS = failures-to-maintain sets. Symbols used for the statistical statistical comparison: ns = non statistically significant, $p > 0.05$; * = $p < 0.05$; ** = $p < 0.01$; *** = $p < 0.001$.

| | CC | TE | PE | NPE | FMS |
|---|---|---|---|---|---|
| **Control** | $6.0 \pm 0.0$ | $12.35 \pm 4.14$ | $6.06 \pm 1.92$ | $6.29 \pm 2.97$ | $0.59 \pm 0.69$ |
| **EPM** | $6 \pm 0.0$ (ns) | $11.76 \pm 3.52$ (ns) | $5.35 \pm 1.81$ (ns) | $6.41 \pm 2.45$ (ns) | $0.24 \pm 0.55$ (ns) |
| **DM** | $0.88 \pm 0.9$ (***) | $47.65 \pm 6.39$ (***) | $18.88 \pm 3.25$ (***) | $28.76 \pm 4.36$ (***) | $2.71 \pm 1.77$ (***) |
| **IM** | $6 \pm 0.0$ (ns) | $11.47 \pm 3.63$ (ns) | $5.59 \pm 1.94$ (ns) | $5.88 \pm 2.89$ (ns) | $0.59 \pm 0.69$ (ns) |
| **VLM1** | $6.0 \pm 0.0$ (ns) | $20.41 \pm 8.35$ (**) | $9.76 \pm 4.35$ (**) | $10.65 \pm 5.47$ (**) | $0.18 \pm 0.38$ (*) |
| **VLM2** | $0.06 \pm 0.24$ (***) | $54.71 \pm 4.08$ (***) | $19.41 \pm 3.57$ (***) | $35.29 \pm 2.82$ (***) | $0.88 \pm 0.83$ (ns) |
| **VLM3** | $6 \pm 0.0$ (ns) | $11.94 \pm 4.25$ (ns) | $5.35 \pm 2.19$ (ns) | $6.59 \pm 3.16$ (ns) | $0.29 \pm 0.57$ (ns) |
| **VLMG** | $0.12 \pm 0.32$ (***) | $60.82 \pm 4.66$ (***) | $21.47 \pm 4.42$ (***) | $39.35 \pm 5.36$ (***) | $1.24 \pm 0.94$ (*) |

Table S3: Mean and standard deviation of the behavioural indices of the control model and the seven lesioned models. The asterisks indicate the significance (two-tail t-tests) of the statistical difference between the lesioned models with respect to the control model. CC = completed categories, TE = total errors, PE = perseverative errors, NPE = non perseverative errors, FMS = failures-to-maintain sets. Symbols used for the statistical comparison: ns = non statistically significant, $p < 0.05$; * = $p < 0.05$; ** = $p < 0.01$; *** = $p < 0.001$.

**Lesions**

Figure S9 shows how the model was lesioned.

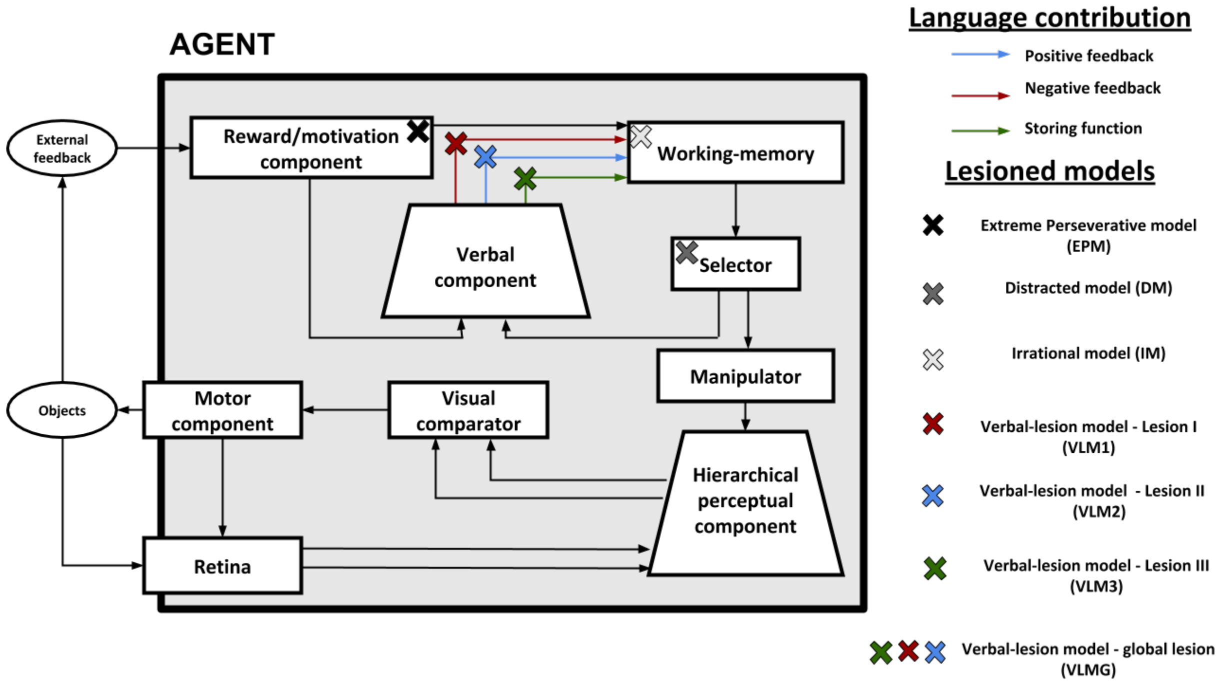


Figure S9: Graphical overview of lesions that we applied to the control model to produce the specific seven lesioned models.

**Internal functioning of the model**

Figure S10 shows the activation of the WM units in different conditions.

**Verbal-lesion model 1 (lesion of language negative feedback processing)**

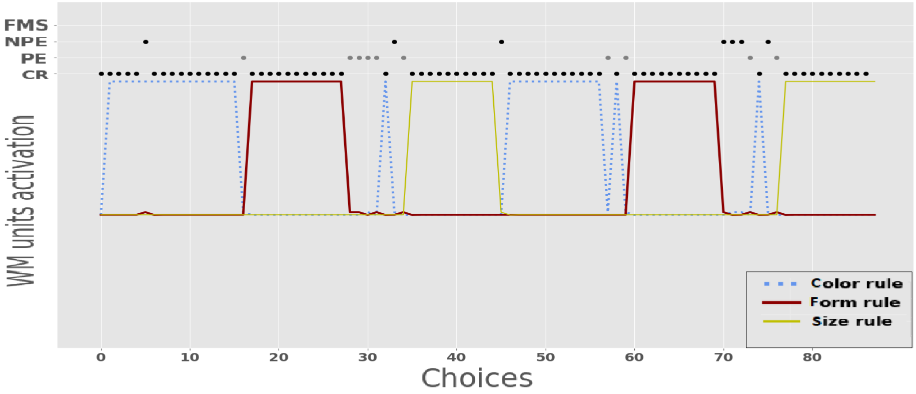


**Verbal-lesion model 2 (lesion of language positive feedback processing)**

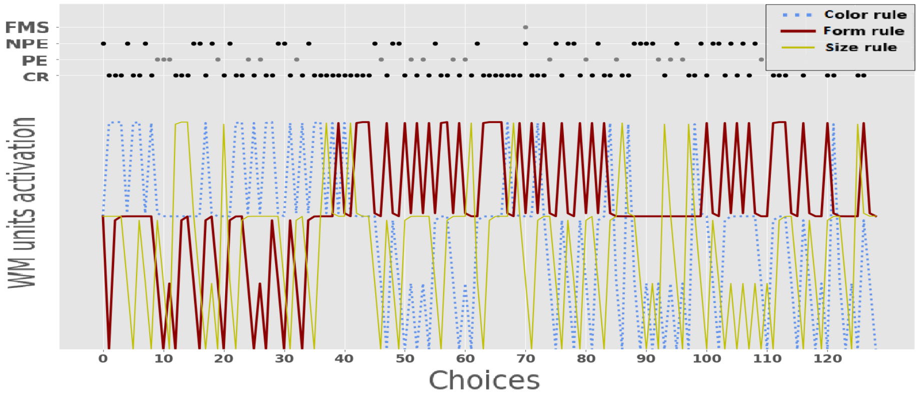


**Global verbal-lesion model (lesion of all language functions)**

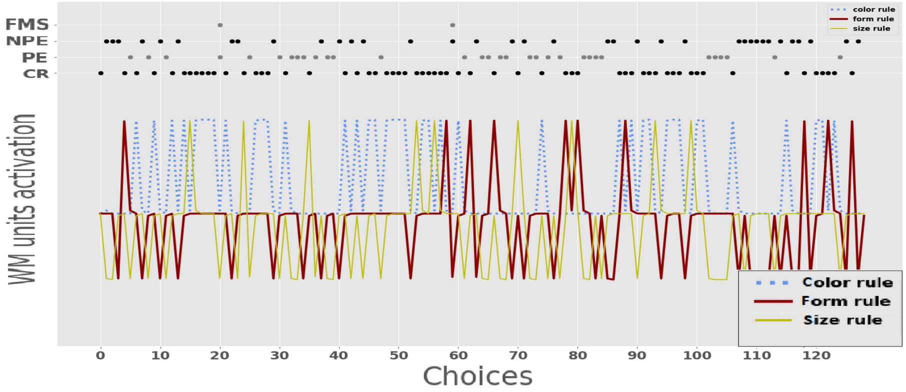


Figure S10: Internal functioning of the three models with lesions affecting different functions of the inner-speech component (Verbal-lesion model 1, Verbal-lesion model 2, Global verbal-lesion model). Each line in the graphs shows the activation of a working-memory unit representing a tendency to choose a specific sorting rule between the three possible rules. The dots at the top of graphs indicate single instances of correct responses (CR) or errors (PE, NPE, FMS).

## References

[1] Van Geit W, De Schutter E, Achard P. Automated neuron model optimization techniques: a review. Biological cybernetics. 2008 Nov;99:241–251.

[2] Baldo JV, Dronkers NF, Wilkins D, Ludy C, Raskin P, Kim J. Is problem solving dependent on language? Brain and language. 2005;92(3):240–250.

[3] Hinton GE, Osindero S, Teh YW. A fast learning algorithm for deep belief nets. Neural computation. 2006;18(7):1527–1554.

[4] Hinton GE. A practical guide to training restricted Boltzmann machines. In: Neural networks: Tricks of the trade. Springer; 2012. p. 599–619.

[5] McClelland JL, Rumelhart DE, Group PR, et al. Parallel distributed processing. Explorations in the Microstructure of Cognition. 1986;2:216–271.

[6] Baldassarre G, Lord W, Granato G, Santucci VG. An embodied agent learning affordances with intrinsic motivations and solving extrinsic tasks with attention and one-step planning. Frontiers in Neurorobotics. 2019;13(45).
